# Relationship between tumor mutational burden, gene mutation status, and clinical characteristics in 340 cases of lung adenocarcinoma

**DOI:** 10.1002/cam4.4781

**Published:** 2022-05-06

**Authors:** Kai Ma, Fengxiang Huang, Yin Wang, Yan Kang, Qilong Wang, Jiaqi Tang, Panfeng Sun, Jiaojiao Lou, Ruiping Qiao, Jiming Si, Jian Cao, Lijun Miao

**Affiliations:** ^1^ Department of Respiratory and Critical Care Medicine First Affiliated Hospital of Zhengzhou University Zhengzhou China; ^2^ Berry Oncology Corporation Beijing China

**Keywords:** immune checkpoint inhibitors, lung adenocarcinoma, tumor mutational burden (TMB)

## Abstract

Tumor mutational burden (TMB) is an emerging predictive marker of response to immune checkpoint inhibitor therapies. We evaluated the correlation between clinical indicators and high‐throughput sequencing results and TMB in lung adenocarcinoma patients, with the aim of finding simpler and more economical factors as surrogate markers for TMB.

The medical records, next‐generation sequencing data, and immunohistochemistry results of 340 lung adenocarcinoma patients who were admitted to the First Affiliated Hospital of Zhengzhou University between 2019 and 2020 were collected. The mutated genes were screened for, and the obtained mutated genes were subjected to functional enrichment analysis using R software. A protein–protein interaction (PPI) network was also constructed, and significant modules in the network were identified. Gene Ontology (GO) analyses were performed for the core genes. Univariate and multivariate correlation analyses were performed to judge the correlation between gene mutations and TMB.

Genes with a junction mutation rate >1 were selected to construct PPI network and 13 high‐connection core genes were screened. The results of GO enrichment analysis showed that the biological processes related to mutant core genes mainly included mitotic cell cycle and cell aging. Subsequently, *ATM* (*p* = 0.006) and *PIK3CA* (*p* = 0.008) mutation positivity were identified by univariate and multivariate correlation analysis, while *TP53* (*p* = 0.003) and *EGFR* (*p* = 0.008) mutation negativity were significantly associated with elevated TMB.

The results of this study demonstrate that *ATM‐* and *PIK3CA*‐positive and *EGFR*‐negative mutation status are strongly associated with high levels of TMB and have the potential to be predictive biomarkers of response to immune checkpoint inhibitors in lung adenocarcinoma patients.

## Graphical abstract

Core genes mutated in lung adenocarcinoma are involved in the regulation of the cell cycle. Age, gender, smoking history, pathological stage, and lymph node infiltration are associated with TMB in patients with lung adenocarcinoma. Mutations in EGFR, ATM, and PIK3CA may provide new ideas for predicting TMB in lung adenocarcinoma patients.

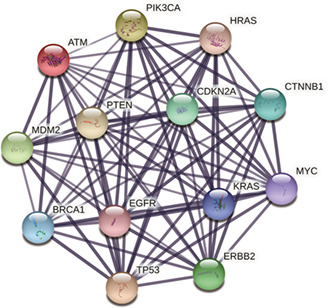

## 1 INTRODUCTION

Lung adenocarcinoma (LUAD) is a type of malignant non‐small cell lung cancer originating from the glandular epithelium of the bronchus mucosa, accounting for about 45% of all lung cancer. Its incidence rate and mortality rate show an increasing trend.^1^ Conventional therapies for LUAD include surgical resection, radiotherapy, and chemotherapy. In recent years, immunotherapy of cancers including LUAD has attracted increasing attention.^2^

Cancer is caused by the accumulation of somatic mutations that lead to the expression of neoantigens,^3^ which can normally be used by the immune system to recognize and clear tumor cells from the tumor microenvironment. However, to survive and grow, tumor cells can adopt different strategies to suppress the body's immune system so that it becomes unable to kill tumor cells, thereby surviving all stages of the antitumor immune response. Under normal physiological conditions, immune checkpoints are critical for maintaining self‐tolerance and preventing autoimmunity, and can also protect tissues from damage when the immune system responds to pathogen infection. However, tumors may interact with such signals, thereby promoting immune escape.^4^ One of the most promising approaches to activate therapeutic antitumor immunity is the blockade of immune checkpoints, which can induce the proliferation and activation of immune cells and fight against cancer cells.^5^

Immune checkpoint inhibitors are currently being widely used to treat cancer. Studies have reported that programmed cell death ligand 1 (PD‐L1) and tumor mutational burden (TMB) are independent predictors of response to immunotherapy in patients with non‐small cell lung cancer (NSCLC),^6^, ^7^ so both PD‐L1 and TMB are promising as biomarkers for the effectiveness of immune checkpoint inhibitors. The use of PD‐L1 expression as a biomarker has been widely studied. In general, the remission rate of anti‐PD‐1/PD‐L1 therapy in patients with PD‐L1‐positive tumors is significantly higher than that of PD‐L1‐negative patients.^8^ However, in the tumor microenvironment, PD‐L1 is expressed in both tumor cells and non‐tumor cells.^9^ The detection of the PD‐L1 expression level is only suitable for patients receiving PD‐1/PD‐L1‐blocking therapy, not for other types of immunotherapies. However, TMB can predict the prognosis of many types of tumors after anti‐PD‐1/PD‐L1 immunotherapy.^10^ TMB is a new predictor of immunosuppressive response. Most studies on TMB have evaluated the relationship between TMB and immunosuppressive response, but the related clinical features have not been well documented. Therefore, in this study, we evaluated the correlation between TMB and clinicopathological features in patients with LUAD to find more convenient factors as alternative markers of TMB.

## 2 METHODS

### 2.1 Sample collection

Medical record data, next‐generation high‐throughput sequencing data (NGS), and immunohistochemical results of 340 LUAD patients who were admitted to the First Affiliated Hospital of Zhengzhou University from 2019 to 2020 and who were definitively pathologically diagnosed by lung puncture were collected. The corresponding specimens were lung puncture tissues. Patient enrollment and genomic studies were conducted in accordance with the Declaration of Helsinki. The study was approved by the ethics committee of the First Affiliated Hospital of Zhengzhou University. All patients provided oral and written informed consent for sample acquisition for research purposes. The inclusion criteria were: (1) immunohistochemical results were examined by pathologists and diagnosed as LUAD; (2) no history of other malignant tumors; and (3) not in receipt of chemotherapy or immunotherapy prior to diagnosis. The exclusion criteria were: (1) combination with other lung diseases; (2) combined disease development with neovascularization, such as diabetes or rheumatoid arthritis; (3) pregnant or lactating patients; and (4) patients with incomplete clinical data. All enrolled samples were examined by pathologists to determine the histological subtype and TNM stage. In total, there were 173 men and 167 women, 155 cases were younger than 65 years old and 185 cases were older than 65 years old. There were 104 patients with a history of smoking and 236 patients that did not smoke. There were 45 cases of stage I disease and 295 cases of stages II–IV disease.

### 2.2 Sample processing and DNA extraction

Fixation of lung puncture tumor tissue with formalin followed by paraffin embedding (FFPE).Genomic DNA was extracted from each FFPE sample using the GeneRead DNA FFPE Kit (Qiagen).

### 2.3 NGS‐based gene panel test

For pre‐library preparation, purified genomic DNA was first fragmented into DNA pieces around 200 bp in length using enzymatic method. After end‐repairing, A‐tailing, T‐adaptors were ligated on both ends, followed by universal primer‐mediated amplification. The purified pre‐library was hybridized with a customized biotin probe panel (the 457 genes panel, BerryoncoPan, Berryoncology)^11^ to capture target DNA fragments. Oncogenes in the probe library were referenced from Catalog of Somatic Mutations in Cancer, the Cancer Genome Atlas, OncoKB, and the Oncomine database oncogene database. The captured DNA fragments were amplified using universal primers and the products were purified to obtain the final library. Paired‐end multiplex samples were sequenced using the NovaSeq 6000 sequencing platform (Illumina). Each sample was sequenced to a depth of around 2000×. The resulting sequences were trimmed, low quality sequences were filtered, and variant calling was performed. The following variant was filtered: non‐synonymous SNPs, indels, and spliced mutations. The allele frequency of variant (cutoff value ≥3%) was defined as somatic variants. Variant allele frequencies (cutoff value ≥1%) and at least 20 high quality reads were defined as cancer hot spots.

### 2.4 Protein interaction network and module analysis

Protein–protein interaction (PPI) networks were constructed using the STRING database.^12^ Subsequently, significant modules in the PPI network were identified using the Cytoscape MCODE plug‐in, with the parameters set as follows: degree cutoff = 2, node score cutoff = 0.2, and *K*‐core = 2. Subsequently, highly connected genes were taken as core genes in the significant module.

### 2.5 The calculation of TMB

The tumor mutation burden (TMB) was determined by the number of all the non‐synonymous mutation and indel variants per magabase of coding regions. The 457 gene panels cover the coding region of 1,141,951 bp. Hence, TMB was calculated with the number of all the non‐synonymous mutations and indel variants/1.14 Mb.

### 2.6 Enrichment analysis of mutant genes

Filtering of mutation characteristics and screening of mutated genes were done according to the previous studies.^11^ The R package Cluster Profiler in R software was used for Gene Ontology (GO) enrichment analysis to study the biological significance of core mutation genes.^13^ The GO database includes three levels, cellular component, molecular function, and biological process. Here, *p* < 0.05 was considered statistically significant.

### 2.7 Statistical analysis

SPSS 21.0 statistical software was used for data analysis. The chi‐squared test was used to analyze the TMB level and clinicopathological parameters of LUAD patients. The Mann–Whitney *U* test was used to compare TMB and gene mutation. Multivariate linear regression analysis was used for correlation analysis. *p* < 0.05 was considered statistically significant.

## 3 RESULTS

### 3.1 Gene mutation frequency in LUAD

**FIGURE 1:**
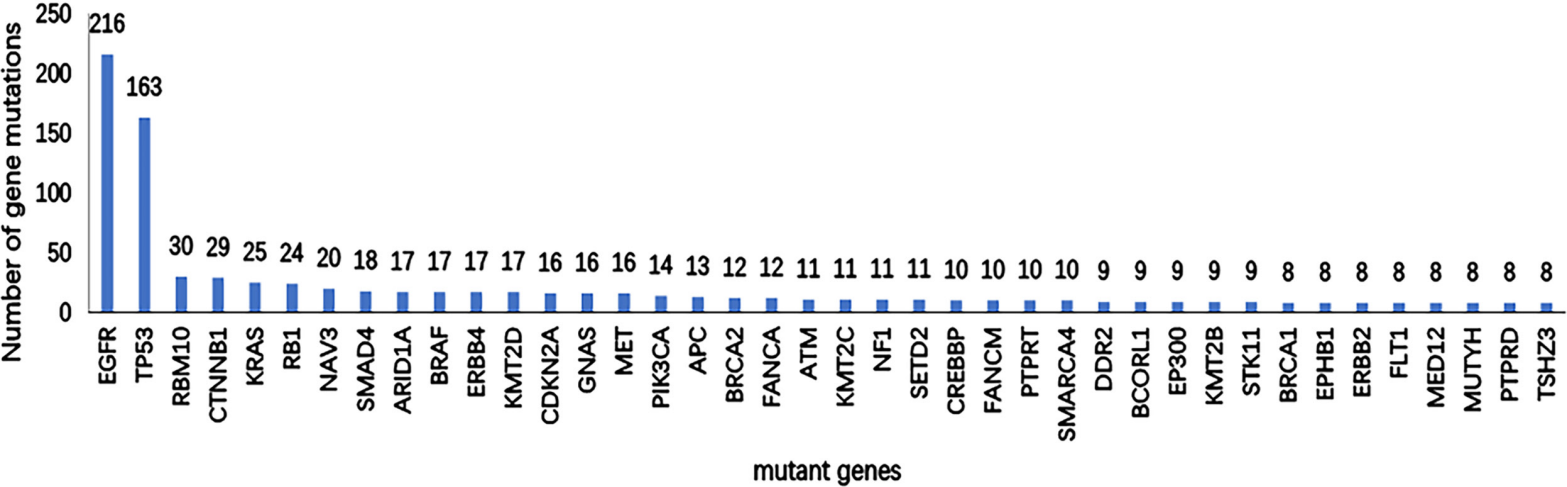
Mutated genes and frequencies in 340 LUAD patients. Genes with the highest mutation frequency are shown here

Gene mutation were analyzed by Illumina sequencing platform and target probe capture technology. The panel of this platform allows the simultaneous evaluation of the mutation status of 457 tumor‐associated genes (BerryoncoPan, Berryoncology). We screened genes with a mutation frequency >1, and the mutation frequencies are shown in Figure 1.

### 3.2 PPI network construction and analysis

**FIGURE 2:**
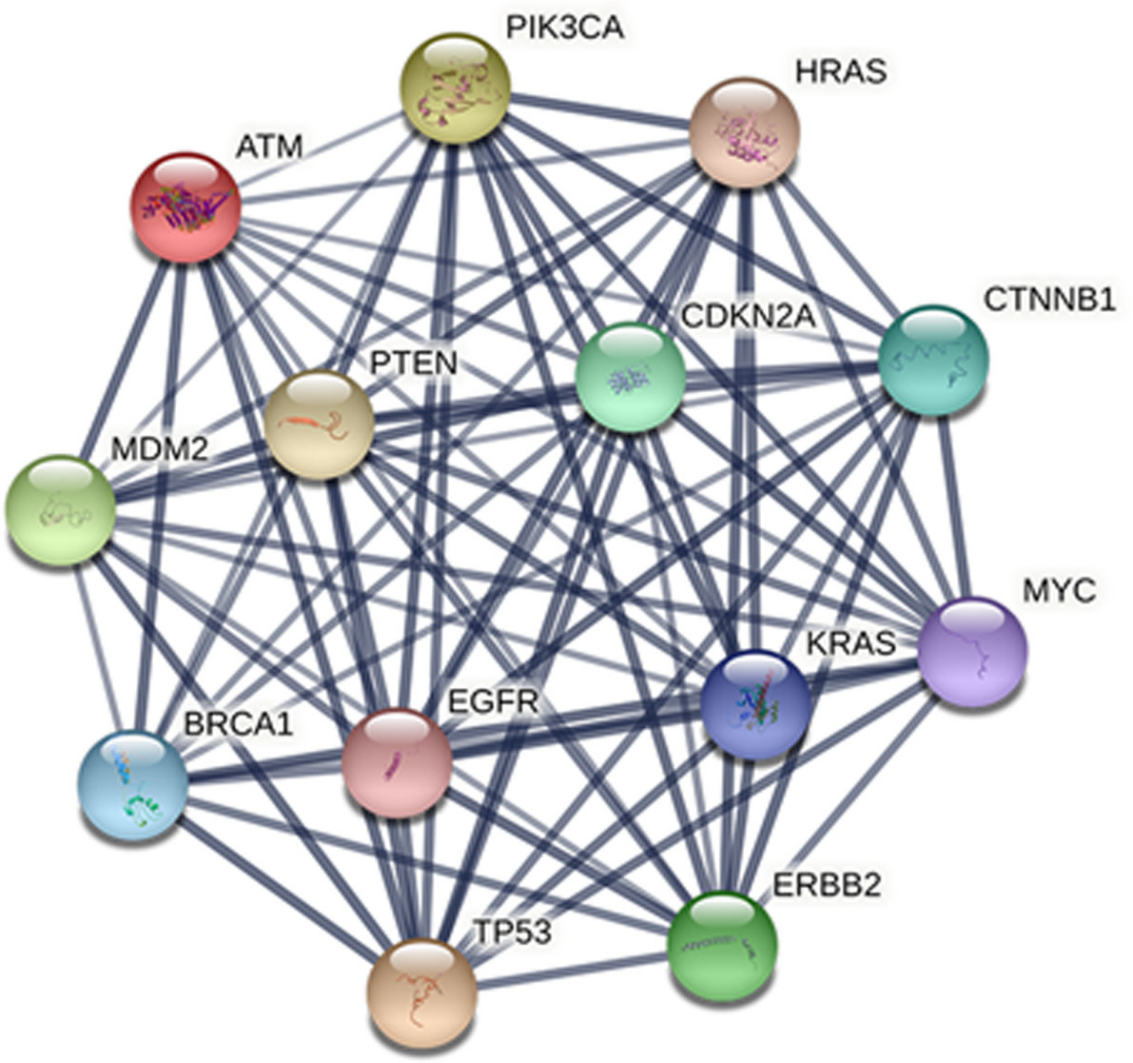
PPI network of 13 core genes

After acquiring data on the differential expression gene interactions using the online database STRING, the PPI network diagram was completed using the MCODE plug‐in in Cytoscape software, and significant modules were screened out by taking an MCODE score value >30 (Figure 2). Subsequently, PPI data were ranked by degree to select 13 genes with high connectivity: *TP53*, *ATM*, *PTEN*, *BRCA1*, *HRAS*, *KRAS*, *PIK3CA*, *MYC*, *CDKN2A*, *CTNNB1*, *MDM2*, *EGFR*, and *ERBB2* (Table 1), which were the core genes.

**TABLE 1:** Connectivity and mutation rate of core mutated genes in the PPI network

Genes	Degree	Mutation rate
TP53	64	47.9%
ATM	58	3.2%
PTEN	57	2.1%
BRCA1	56	2.4%
HRAS	55	0.6%
KRAS	55	7.4%
PIK3CA	55	4.1%
MYC	54	0.3%
CDKN2A	52	4.7%
CTNNB1	51	8.5%
MDM2	51	1.1%
EGFR	50	63.5%
ERBB2	50	2.4%

### 3.3 Gene set enrichment analysis

**FIGURE 3:**
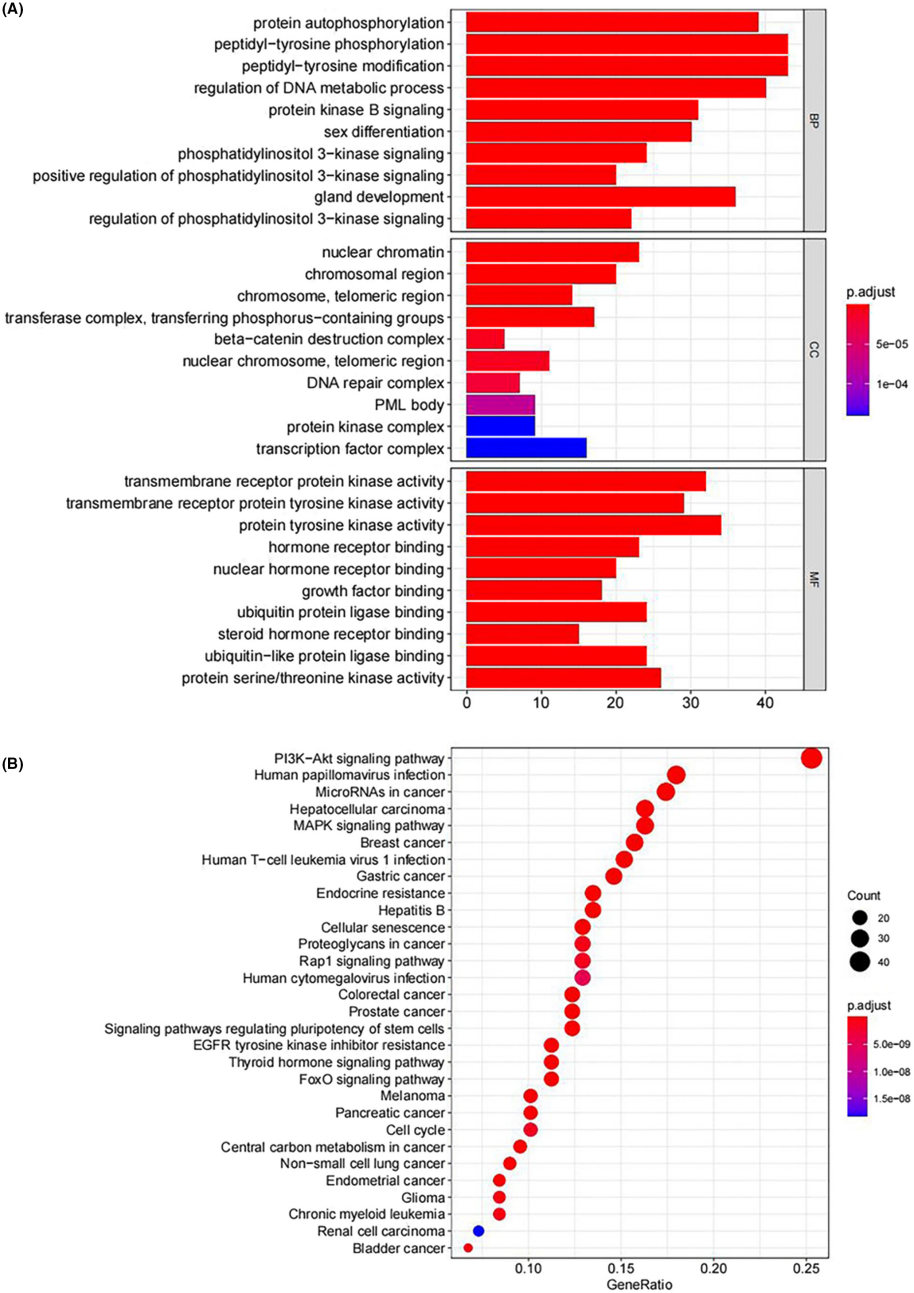
GO enrichment histogram. The abscissa is the number of core genes enriched in the GO analysis, the ordinate is the enriched GO, and the color represents the statistical significance

To better understand the biological functions of the core mutation genes, GO enrichment analyses were performed. The GO enrichment analysis results showed that the biological processes associated with the mutated genes mainly included negative regulation of mitotic cell cycle, cell cycle arrest, cell aging, and regulation of DNA metabolic process. The molecular functions mainly included 1‐phosphatidylinositol‐3‐kinase activity, phosphatidylinositol 3‐kinase activity, phosphatidylinositol kinase activity, protein phosphatase binding, and ubiquitin protein ligase binding. In addition, these mutated genes were also closely associated with cellular components such as basal plasma membrane, basolateral plasma membrane, basal part of cell, extrinsic component of membrane, membrane raft, membrane microdomain, apical plasma membrane, and membrane region (Figure 3).

### 3.4 Relationship between TMB levels and clinicopathological characteristics

Table 2 shows the percentage of patients with high TMB among the 340 LUAD patients. The threshold for high TMB was set to 10 based on previous studies.^11^ It was higher in patients younger than 65 years (65/105 vs. 90/235, *χ* ^2^ = 16.305, *p* < 0.001), male patients (72/105 vs. 101/235, *χ* ^2^ = 19.020, *p* < 0.001), smokers (51/105 vs. 53/235, *χ* ^2^ = 21.139, *p* < 0.001), and patients with stage II–IV (92/105 vs 173/235, *χ* ^2^ = 5.708, *p* = 0.006), and N1‐3 (77/105 vs. 134/235, *χ* ^2^ = 10.081, *p* = 0.002).

**TABLE 2:** Relationship between TMB and clinicopathological characteristics

Clinicopathological characteristics	number	TMB level		*χ* ^2^	*p*
		Low TMB	High TMB		
Age		235	105		
≥65	185	145	40	16.305	<0.001
<65	155	90	65		
Gender					
Male	173	101	72	19.020	<0.001
Female	167	134	33		
Smoking					
Yes	104	53	51	21.139	<0.001
No	236	182	54		
Stage					
I	45	38	7	5.708	0.011
II–IV	295	197	98		
T					
T1‐2	206	148	58	1.821	0.110
T3‐4	134	87	47		
N					
N0	116	93	23	10.081	0.001
N1‐3	224	142	82		
M					
M0	132	95	37	0.822	0.216
M1	208	140	68		

### 3.5 Relationship between core genes and TMB

Univariate correlation test results indicated that of the core genes, *ATM* (*p* = 0.006) and *PIK3CA* (*p* = 0.008) mutation positivity were significantly associated with elevated TMB, while *TP53* (*p* = 0.003) and *EGFR* (*p* = 0.008) mutation negativity were significantly associated with elevated TMB (Table 3).

**TABLE 3:** Relationship between TMB and core genes

	*N*	Mean	95%CI	*p*
TP53				
+	163	5.1775	4.6550–5.7038	0.003
−	177	5.3329	4.4239–6.2437	
ATM				
+	11	10.1909	5.8400–14.8874	0.006
−	329	5.0935	4.5462–5.6248	
PTEN				
+	7	6.2557	3.7528–8.6342	0.168
−	333	5.2375	4.6827–5.7790	
BRCA1				
+	8	7.7738	4.3800–11.6352	0.08
−	332	5.1987	4.6397–5.7482	
HRAS				
+	2	11.8250	8.7600–14.8900	0.054
−	338	5.2196	4.6696–5.7453	
KRAS				
+	25	7.3920	4.8762–10.1858	0.119
−	315	5.0891	4.5425–5.6402	
PIK3CA				
+	14	9.1800	5.5717–13.9133	0.008
−	326	5.0900	4.5554–5.6064	
MYC				
+	1	4.3800	4.3800–4.3800	0.865
−	339	5.2610	4.7306–5.8124	
CDKN2A				
+	16	7.6625	5.8387–9.3706	0.002
−	324	5.1397	4.5742–5.6958	
CTNNB1				
+	29	6.4021	4.9457–7.9788	0.005
−	311	5.1518	4.5875–5.7167	
MDM2				
+	4	10.2900	3.500–25.4000	0.130
−	336	5.1985	4.6432–5.7111	
EGFR				
+	216	4.3901	3.8777–4.8967	0.014
−	124	6.7710	5.6243–8.0017	
ERBB2				
+	8	4.5998	2.6300–5.8167	0.901
−	332	5.2743	4.7019–5.8167	

### 3.6 Relationship between TMB core genes, clinicopathological characteristics, and TMB

Multiple linear regression was used to predict TMB according to the clinicopathological parameters and core mutation genes. As shown in Table 4, the regression model had statistical significance (*F* = 6.013, *p* < 0.001, adjusted *R* ^2^ = 0.168). The influence of all four independent variables included in the model on TMB was statistically significant (*p* < 0.05). The results are shown in Table 4. We deduced the equation for predicting TMB:

**TABLE 4:** Relationship between TMB core genes, clinicopathological characteristics, and TMB

	*n*	*R*	*p*
Age	340	0.577	0.287
Gender	340	1.396	0.044
Smoking	340	1.315	0.089
Stage	340	0.499	0.454
N	340	−0.707	0.448
EGFR	340	−1.443	0.013
TP53	340	−0.134	0.803
ATM	340	3.250	0.034
PIK3CA	340	4.102	0.002
CDKN2A	340	1.951	0.121
CTNNB1	340	1.298	0.172

TMB = 4.059 + 1.396 × (male: 1, female: 0) − 1.443 × (EGFR+: 1, EGFR−: 0) + 3.250 × (ATM+: 1, ATM−: 0) + 4.102 × (PIK3CA+: 1, PIK3CA−: 0).

## 4 DISCUSSION

The results showed that a high level of TMB was strongly correlated with *ATM* and *PIK3CA* mutation positivity and *EGFR* mutation negativity. The *ATM* gene is a pathogenic mutation gene on chromosome 11q22.3, which is one of the frequently mutated genes in cancer. ATM protein is a member of the PI3K‐like protein kinase family, which is involved in genome stability, cell response to DNA damage, and cell cycle control.^14^ The main function of ATM protein is to participate in the regulation of the cell cycle and recognize and repair DNA damage.

PI3K is a lipid kinase that regulates important signaling pathways in tumorigenesis, including cell proliferation, adhesion, survival, and motility.^15^, ^16^ PI3K consists of the p85 regulatory sub‐unit and p110 catalytic sub‐unit. The latter is encoded by three genes, *PIK3CA*, *PIK3CB*, and *PIK3CD*. *PIK3CA* mutation is the most common gene mutation in cancer and is an independent risk factor for affecting the overall survival (OS) and progression‐free survival (PFS) in NSCLC patients.^4^, ^17^

In contrast to patients with *EGFR* mutations who are insensitive to PD‐1/L1 inhibitors (HR = 1.09, *p* = 0.51), *EGFR* wild‐type patients were shown to benefit from PD‐1/L1 inhibitors (HR = 0.73, *p* < 0.00001). The same study provided evidence of a correlation between *EGFR* gene mutations and a noninflammatory tumor microenvironment with immune tolerance and weak immunogenicity, contributing to the poor response of NSCLCs to PD‐1 blockade, and found that *EGFR* mutation (*EGFR*‐mut) was negatively correlated with TMB.^18^ Other studies have also demonstrated an inverse correlation between TMB and prognosis with targeted therapies in *EGFR*‐mut lung cancer patients exhibiting poor responses to immunotherapy.^19^, ^20^

Studies have reported that smoking is significantly associated with high TMB levels.^21^ The TMB level was significantly higher in men than in women.^22^ A significant inverse correlation was found between age and TMB level in *TP53*‐mut LUAD patients.^23^ This is consistent with the results of the present study. TMB can predict the prognosis of many types of tumors after anti‐PD‐1/PD‐L1 immunotherapy.^10^ Patients with high TMB have longer OS,^10^ PFS,^21^ and objective response rate (ORR)^24^ after receiving immunotherapy compared with those with low TMB. Studies that counted the data of durable clinical benefit (DCB) and TMB in patients who received immunotherapy concluded that TMB was significantly higher in patients who experienced DCB than those who did not.^21^

Tumors with a large number of non‐synonymous somatic mutations identified by whole‐exome sequencing (WES) are more likely to respond to checkpoint blocking immunotherapy. Theoretically, these tumors will have a higher diversity of new antigens, and when the inhibition of PD‐1/PD‐L1 is blocked, the immune response can be triggered.^25^, ^26^ Melanoma and NSCLC showed the strongest response to PD‐1/PD‐L1 blockade, both of which had higher TMB.^27^ As assessed by WES, a high burden of non‐synonymous mutations was associated with ORR, long‐term clinical benefit, and PFS in patients receiving anti‐PD‐1/PD‐L1 treatment. However, WES is expensive and time‐consuming, so it is not a routine clinical examination.^28^ Targeted panel sequences focusing on cancer‐related genes are also available. Targeted panel sequencing analysis can provide an alternative marker for TMB and may be easier to introduce into clinical application.^29^ In addition, the cost of using WES to determine TMB is about 10 times that of using cancer hot spot panel sequencing to determine *EGFR* and *ATM* status. Therefore, a simpler and more economical method is needed to estimate TMB and predict the response to checkpoint blockade. It may be a useful alternative to predict TMB by analyzing specific gene changes, such as *EGFR*.

Because TMB is considered a biomarker of response to immune checkpoint inhibitors, analysis of *ATM*, *PIK3CA*, and *EGFR* mutations may provide a rapid and simple way to predict TMB. Although the correlation of these parameters with TMB in this predictive model is not high enough, biomarkers involving the combination of multiple factors may become more important in the future. Further studies are needed to confirm our results and assess the value of *ATM*, *PIK3CA*, and *EGFR* mutations as predictive biomarkers of response to immune checkpoint inhibitors in patients with NSCLC.

## CONFLICT OF INTEREST

Yin Wang and Jian Cao are employees of Berry Oncology.

## AUTHOR CONTRIBUTIONS

Kai Ma, Yin Wang, and Fengxiang Huang designed the experiments. Jiaqi Tang, Jiaojiao Lou, and Panfeng Sun collected data. Qilong Wang, Ruiping Qiao and Panfeng Sun analyzed the data. Kai Ma, Jiming Si, and Fengxiang Huang conducted statistical analysis. Kai Ma, Yan Kang, and Jian Cao wrote the manuscript. Lijun Miao revised the manuscript.

## ETHICS APPROVAL

Patient enrollment and genomic studies were conducted in accordance with the Declaration of Helsinki. The study was approved by the ethics committee of the First Affiliated Hospital of Zhengzhou University.

## PATIENT CONSENT STATEMENT

Clinical information was collected with the informed consent of each patient.

## Data Availability

The data used to support the findings of this study are available from the corresponding author upon request.
